# Oral vs Intravenous Fluid Therapy in Emergency Department Patients: Protocol for a Randomized Controlled Feasibility and Noninferiority Trial

**DOI:** 10.2196/95459

**Published:** 2026-09-30

**Authors:** Peter Biesenbach, Mette Rahbek Kristensen, Mikkel Brabrand, Christian Backer Mogensen, Line Emilie Laugesen

**Affiliations:** 1 Department of Emergency Medicine Esbjerg Hospital University of Southern Denmark Esbjerg, South Denmark Denmark; 2 Department of Emergency Medicine Odense University Hospital University of Southern Denmark Odense, South Denmark Denmark; 3 Department of Emergency Medicine Hospital Sønderjylland University of Southern Denmark Aabenraa, South Denmark Denmark

**Keywords:** emergency medicine, fluid therapy, oral fluid, resuscitation, fluid methods, rehydration solutions, infusions, intravenous, crystalloid solutions, randomized controlled trial, hypovolemia

## Abstract

**Background:**

Intravenous fluid therapy is widely used in emergency departments (EDs) to treat hypovolemia and hypotension. However, its hemodynamic effects are often modest and short-lived. Intravenous fluid therapy requires a peripheral venous catheter, which increases the risk of complications such as catheter failure and phlebitis. Oral fluid therapy represents a simple, noninvasive alternative that has been shown in physiological studies to expand plasma volume and increase blood pressure. No randomized trial has compared oral with intravenous fluid therapy in a general adult ED population. Evidence regarding the feasibility of randomized comparisons of fluid administration modes and whether oral fluid administration can reliably deliver the prescribed volume remains limited.

**Objective:**

This study aims to evaluate the feasibility of conducting a randomized controlled trial of oral vs intravenous fluid therapy in ED patients requiring fluid replacement and to determine whether oral fluid therapy is noninferior to intravenous fluid therapy in achieving the prescribed fluid volume.

**Methods:**

This investigator-initiated, multicenter, open-label randomized controlled feasibility and noninferiority trial will enroll 250 adult ED patients prescribed ≥1000 mL of intravenous crystalloid therapy. Participants will be randomized 1:1 to receive either oral fluids or intravenous crystalloid. The primary feasibility outcome is protocol adherence, defined as the proportion of randomized participants who receive the allocated route of administration without a major protocol deviation. The primary clinical outcome is the proportion of patients achieving the prescribed fluid volume during the ED stay, censored at 48 hours after randomization and analyzed using a noninferiority framework. Noninferiority of oral fluid therapy will be tested against the null hypothesis that the intravenous proportion exceeds the oral proportion by at least the noninferiority margin of 15 percentage points, using the difference in proportions with a 1-sided 97.5% CI (equivalent to a 2-sided 95% CI) from an exact proportion test. The sample size of 250 will provide 90% power at a 1-sided α of .025 under an assumed proportion of 85% in both arms.

**Results:**

The study was funded by the Region of Southern Denmark in May 2024. Recruitment began in October 2025 at 3 Danish EDs, and 85 participants have been recruited as of September 2026. Recruitment is projected to close in October 2026, with final 30-day follow-up projected for November 2026. No amendments to the protocol have been made. Results are expected to be published in summer 2027.

**Conclusions:**

This trial will evaluate whether a randomized trial comparing intravenous and oral fluid therapy is feasible in ED patients and whether oral fluid therapy can achieve prescribed fluid volumes comparable to those achieved with intravenous therapy. The findings will inform the design of a future definitive effectiveness trial.

**Trial Registration:**

ClinicalTrials.gov NCT07361952; https://clinicaltrials.gov/study/NCT07361952

**International Registered Report Identifier (IRRID):**

DERR1-10.2196/95459

## Introduction

### Background

Intravenous fluid therapy is among the most frequently administered interventions in emergency departments (EDs) and critical care settings [1,2]. It is routinely used to improve organ perfusion in patients with hypotension and hypovolemia; however, the hemodynamic effects of fluid therapy are often modest and short-lived [3]. Evidence guiding fluid therapy strategies remains inconclusive in several key areas. Large clinical trials have failed to demonstrate consistent differences in outcomes between balanced crystalloids and isotonic saline [4,5] as well as between restrictive and liberal fluid strategies [6-8]. Liberal intravenous fluid administration may also contribute substantially to health care resource use [9]. Despite the uncertainty, intravenous fluid therapy remains a cornerstone of ED treatment in acute illness [10,11].

Another aspect of fluid administration has not yet been explored in the ED setting: whether it is at all necessary to administer a fluid bolus intravenously, as an infusion. Intravenous therapy requires a peripheral intravenous catheter, which carries the risk of complications such as occlusion, dislodgement, phlebitis, and bacteremia [12,13]. A large number of ED patients receive peripheral venous catheters despite not strictly requiring them [14,15].

Oral fluid administration represents a simple, noninvasive alternative. Beyond its practicality, oral fluid administration exerts distinct physiological effects. Previous data suggest that oral fluids induce expansion of the circulating plasma volume, recruitment of splanchnic blood volume, peripheral vasoconstriction [16,17], increased cerebral blood flow [18], and increased blood pressure [19]. Collectively, these findings indicate that oral fluid therapy induces a range of cardiovascular responses associated with sympathetic activation, although the precise underlying mechanisms remain incompletely understood [20].

Despite these physiological observations, prospective clinical evidence remains limited. In a randomized crossover trial conducted by our group in healthy participants, oral fluid therapy resulted in a significantly greater and more sustained increase in mean arterial pressure than intravenous fluids [21]. Similarly, a greater increase in blood pressure was also reported in intensive care patients in shock when fluids were administered via a nasogastric tube [22]. A meta-analysis published in 2018 found oral hydration to be as effective as intravenous hydration in preventing contrast-induced nephropathy [23]. A trial showed no difference in outcome for acute pancreatitis between nasojejunal and intravenous fluid therapy [24]. In pediatric populations, a randomized trial demonstrated that oral fluid therapy was not inferior to intravenous fluid therapy [25], and a meta-analysis of 16 randomized controlled trials investigating gastroenteritis reported shorter hospital stays and fewer serious adverse events in the oral group [26].

Despite these findings, no prospective randomized controlled trials have compared oral and intravenous fluid therapy in adult ED populations. Intravenous fluids are frequently administered to patients who are alert, cooperative, and able to swallow. Yet it remains uncertain whether oral administration can reliably deliver prescribed fluid volumes in routine emergency care. The advantage postulated for oral fluid therapy is not that it delivers fluid better than the intravenous route, but that it delivers fluid at similar rates. Establishing feasibility and exploring noninferiority in volume delivery are therefore necessary steps before conducting a definitive effectiveness trial.

### Objectives

The aim of this study is to evaluate the feasibility of conducting a randomized controlled trial comparing oral and intravenous fluid therapy in ED patients requiring fluid replacement and to determine whether oral fluid therapy is noninferior to intravenous fluid therapy in achieving the prescribed fluid volume during the trial period, defined as the ED stay censored at 48 hours after randomization.

## Methods

### Study Design

This study is an investigator-initiated, multicenter, open-label, randomized controlled feasibility and noninferiority trial. The study protocol is reported in accordance with the SPIRIT (Standard Protocol Items: Recommendations for Interventional Trials) statement [27]. A flow diagram of the study design is shown in Figure 1.

The trial has 2 primary objectives. The primary feasibility objective is to evaluate the feasibility of administering oral fluid therapy in the ED setting, assessed by protocol adherence. The primary clinical objective is to determine whether oral fluid therapy is noninferior to intravenous fluid therapy in achieving the prescribed fluid volume during the trial period, defined as the ED stay censored at 48 hours after randomization. The study is designed as a pragmatic trial to reflect routine clinical practice.

**Figure 1:**
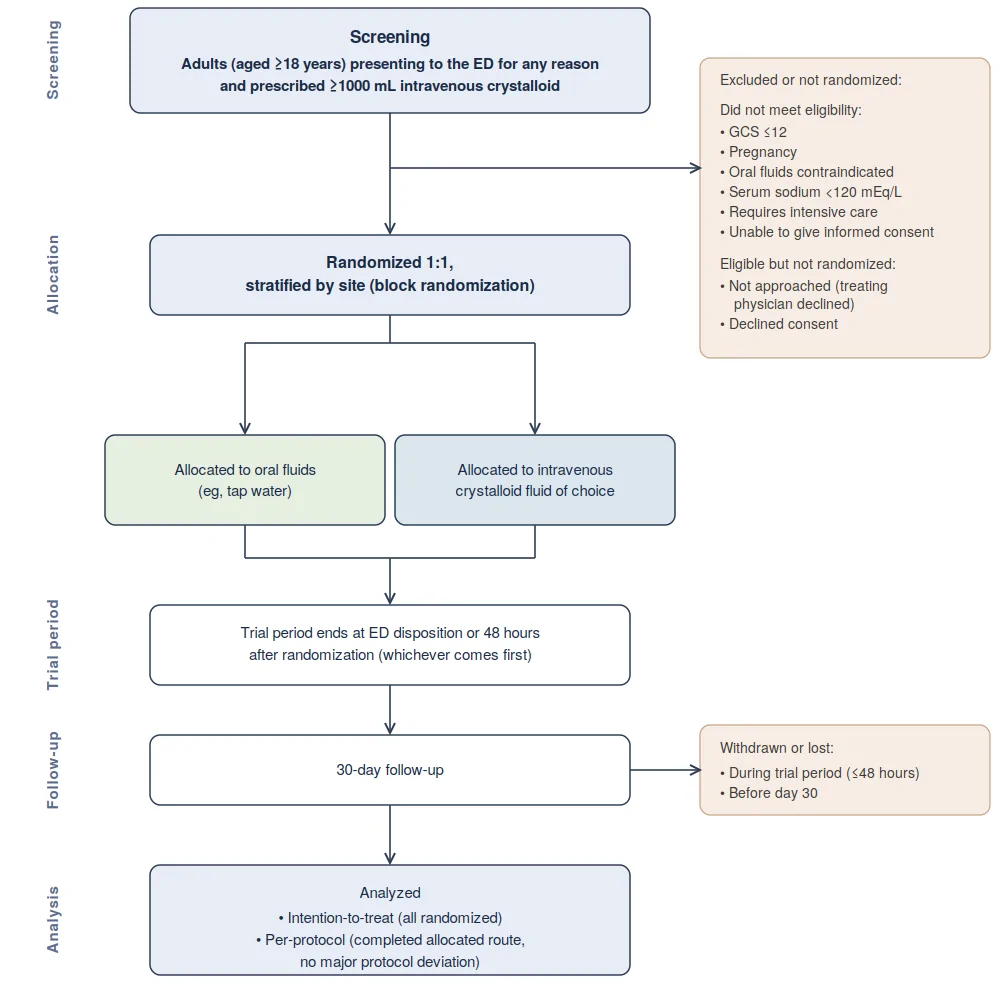
Participant flow through the trial, showing screening, randomization, allocation, the trial period, follow-up, and analysis populations. ED: emergency department; GCS: Glasgow Coma Scale.

### Study Setting

The trial is being conducted at 3 Danish EDs in the Region of Southern Denmark. Participants are recruited from the EDs at the University Hospitals of Aabenraa, Esbjerg, and Odense. The combined catchment area is around 950,000 inhabitants.

Research personnel will recruit participants and collect data. All research personnel will follow standardized protocols for screening and patient inclusion, as well as follow-up and data collection. The study originates from the research unit for emergency medicine in Esbjerg.

### Study Period

Recruitment began in October 2025 and is expected to continue until October 2026.

### Population and Eligibility Criteria

Adults aged ≥18 years presenting to the ED for any reason will be invited to participate in the study if the treating physician prescribes a minimum of 1000 mL of crystalloid intravenous fluid therapy during the ED stay. Only patients able to provide informed consent will be included. A patient can participate in the study only once.

Patients are excluded if they fulfill any of the following criteria: Glasgow Coma Scale score ≤12 (altered mental state prohibiting oral fluid intake), pregnancy, contradictions to oral fluids, hyponatremia <120 mEq/L, or a requirement for treatment in the intensive care unit.

### Recruitment and Informed Consent

Research personnel identify eligible participants presenting to the ED through electronic health records during weekdays between 08:00 AM and 4:00 PM. The treating clinician is contacted as soon as intravenous fluid treatment has been prescribed. If the patient meets the eligibility criteria, research personnel will inform the patient about the study details and invite them to participate in the study. All screened patients are recorded in a screening log, including those meeting an exclusion criterion, those for whom the treating physician declines an approach, and those declining participation, with the reason for noninclusion in each case.

### Assignment of Interventions

Randomization is performed stratified per site using block randomization with a 1:1 allocation to the intervention or control group, using the electronic randomization system accessible through REDCap (Vanderbilt University) via the Open Patient Data Explorative Network (OPEN; Odense University Hospital [OUH]) [28]. After obtaining written consent from the participant, the research staff will create a new record and randomize the patient within the program, automatically assigning the patient to a treatment arm. Personnel who will enroll participants and those who will assign participants to the interventions will not have access to the random allocation sequence.

### Concealment

Due to the type of intervention, it is not possible to blind the allocation of treatment to the participants or study representatives. The biostatistician performing the analysis will be blinded to the allocation.

### Intervention (Oral Fluids)

The treatment consists of clear fluids administered orally of any type (eg, tap water or tea). The fluid temperature will not be specified for the trial. The fluids will be administered via regular single-use cups available in the ED. The bedside nurse encourages the patient to drink if necessary to achieve the prescribed fluid volume. During the trial period, the patient receives any prescribed fluid volume orally over a predefined time frame decided by the attending physician. Patients are allowed to drink additional fluids. Participation in the oral fluid administration arm will be ended in the event of >2 episodes of vomiting refractory to medication.

### Control (Intravenous Fluids)

The type of intravenous fluid is chosen by the physician from the standard repertoire of crystalloid infusions available at the ED. These include Ringer’s lactate, 0.9% sodium chloride, and Ringer’s acetate. The crystalloid infusions are stored at room temperature in the medical storage room or within the patient’s room and are also administered at room temperature. Empty fluid packages are not stored after use. During the trial period, the patient receives any prescribed fluid volume intravenously over a predefined time frame decided by the attending physician. Patients are allowed to drink additional fluids.

### Trial Period

The trial period is limited to the stay in the ED. In the case of discharge to home, admission to the ward, or transfer to another medical facility, the trial period stops. Furthermore, 48 hours after inclusion, the trial period stops, even if the patient is still boarded in the ED. Transport within the ED for any reason, including X-rays, computed tomography imaging, or echocardiography, does not affect participation. In both groups, the total fluid volume as well as the rate of fluid administration during the ED stay will be decided by the physician, and repeat fluid boluses are not limited. The study does not limit additional care, including other types of oral or intravenous fluids such as antibiotics or transfusions.

### Outcomes

#### Overview

All outcomes are summarized in Tables 1 and 2.

**Table 1:** Feasibility outcome measures.

Outcomes		Definitions	Metrics	Time points	
Primary feasibility outcome: protocol adherence		Proportion of participants with no major protocol deviation. Major protocol deviations are defined as follows: (1) enrollment of a participant who met an exclusion criterion, (2) randomization without valid informed consent, or (3) administration of intravenous crystalloid for volume replacement to a participant allocated to the oral arm. Oral intake is permitted in both arms and does not constitute a protocol deviation. Discontinuation of the oral arm under the prespecified stopping rule (>2 episodes of vomiting refractory to medication) is considered protocol-adherent.	Proportion with 95% CI, overall and by site		ED^a^ stay (censored at disposition or 48 hours)
**Secondary feasibility outcomes**					
	Recruitment rate	Number of participants recruited over time at each site, relative to the catchment population	Descriptive (count; rate per site-month)		Enrollment period
	Data collection feasibility	Extent, pattern, and reasons for missing source data	Descriptive (proportion missing by variable, with reasons)		Throughout the study
	Study completion rate	Proportion of participants completing the study through day 30	Descriptive (proportion with 95% CI)		Day 30
	Resource requirements	Time, staff, and financial resources required to conduct the trial	Descriptive		Throughout the study
	Fidelity rate	Consistency of intervention delivery across sites	Descriptive (proportion adherent by site)		ED stay
	Recruitment cascade	Numbers screened, excluded by criterion, not approached at the treating physician’s request, declining consent, randomized, and withdrawn during the trial period or before day 30, with reasons	Descriptive; CONSORT^b^ flow diagram		Screening through day 30

^a^ED: emergency department.

^b^CONSORT: Consolidated Standards of Reporting Trials.

**Table 2:** Clinical outcome measures.

Outcomes		Definitions	Metrics	Time points
Primary clinical outcome: achievement of prescribed fluid volume		Proportion of participants achieving the prescribed fluid volume by the allocated route during the trial period	Risk difference (oral minus IV^a^) with 2-sided 95% CI (exact proportion test); noninferiority will be concluded if the lower limit exceeds −15 percentage points in both the intention-to-treat and per-protocol populations	ED^b^ stay (censored at disposition or 48 hours)
**Secondary clinical outcomes**				
	Fluid received during the ED stay	IV, oral, and total fluid volume received during the entire ED stay	Mean (SD) or median (IQR); *t* test or Mann-Whitney *U* test, according to distribution	ED stay
	Fluid received during the study period	IV, oral, and total fluid volume received during the trial period	Mean (SD) or median (IQR); *t* test or Mann-Whitney *U* test	Trial period (≤48 hours)
	Change in blood pressure	Change in systolic and mean arterial pressure	Mean change with 95% CI; *t* test or Mann-Whitney *U* test	Baseline to 8 hours or discharge, whichever occurs first
	Hospital-free days alive	Days alive and out of hospital, dichotomized as event-free at 30 days	Proportion; chi-square test or Fisher exact test; RR^c^ with 95% CI	Day 30
	Mortality	All-cause mortality	Proportion; chi-square test or Fisher exact test; RR with 95% CI	Day 30
	PIVC^d^ hours	Number of hours with a peripheral IV catheter in situ	Median (IQR); Mann-Whitney *U* test	ED stay through PIVC removal
	PIVC avoidance	Proportion of participants without any PIVC during the study period	Proportion; chi-square test or Fisher exact test; RR with 95% CI	Trial period (≤48 hours)
	Acute kidney injury	Acute kidney injury according to RIFLE^e^ criteria within 48 hours	Proportion; chi-square test or Fisher exact test; RR with 95% CI (exploratory safety)	Within 48 hours
	Severe electrolyte derangement	Sodium <120 or >160 mmol/L, potassium <3 or >6 mmol/L, or chloride <90 or >110 mmol/L within 48 hours	Proportion; chi-square test or Fisher exact test; RR with 95% CI (exploratory safety)	Within 48 hours
**Substudy outcomes**				
	Continuous hemodynamics (substudy 1)	Blood pressure, cardiac output, stroke volume, pulse, and systemic vascular resistance index measured noninvasively using Edwards HemoSphere; 10 participants per arm	Descriptive (summary statistics); not powered for comparison	First 4 hours of fluid therapy
	Health-related quality of life (substudy 2)	36-item Short Form Health Survey, comprising 8 domains; 10 participants per arm	Descriptive (domain scores); not powered for comparison	Day 30
	Anxiety and depressive symptoms (substudy 2)	Hospital Anxiety and Depression Scale, comprising 14 items; 10 participants per arm	Descriptive (subscale scores); not powered for comparison	Days 7 and 30

^a^IV: intravenous.

^b^ED: emergency department.

^c^RR: relative risk.

^d^PIVC: peripheral intravenous catheter.

^e^RIFLE: risk, injury, failure, loss of kidney function, and end-stage kidney disease.

#### Substudy 1: Continuous Blood Pressure Monitoring

In 10 patients receiving the intervention and 10 patients from the control group, we will measure hemodynamic parameters (blood pressure, cardiac output, stroke volume, pulse, and systemic vascular resistance index) continuously via a noninvasive device (Edwards HemoSphere; Becton, Dickinson and Company) over the first 4 hours of fluid therapy.

#### Substudy 2: Patient Satisfaction

Ten patients from each group, for a total of 20 patients, will receive an electronic questionnaire measuring quality of life on day 30, the 36-item Short Form Health Survey, a patient-reported questionnaire that covers 8 health domains and has been validated in a Danish general-population sample [29]. Patients are also provided with the Hospital Anxiety and Depression Scale to complete on days 7 and 30, a self-assessment scale for detecting states of depression and anxiety in the hospital setting with established validity in patients with medical illness [30]. All questionnaires will be delivered to the patient through electronic or standard mail if consent is given.

### Data Collection Methods

Data collection will be carried out by dedicated and trained research personnel. Data collected will be entered by research personnel into the electronic database REDCap via OPEN at OUH, and/or uploaded and stored on a secure SharePoint site. Patients who are screened but not randomized are not entered into the trial database; only the screening log entry and the reason for noninclusion are retained. Participants may withdraw at any time without giving a reason. For participants who withdraw after randomization, data collected up to the point of withdrawal are retained and analyzed unless deletion is requested, in which case the record is deleted.

### Data Collected

A schedule of measurements is provided in Figure 2. Information necessary for assessing eligibility of potential participants prior to obtaining informed consent is disclosed to the investigator in accordance with applicable data protection regulations. For screening, the following parameters are assessed without patient consent: social security number, name, admission blood tests, pregnancy test, prescriptions ordered by the physician, and vital signs (systolic and diastolic blood pressure, mean arterial pressure, heart rate, peripheral oxygen saturation, Glasgow Coma Scale score, and ear temperature).

After consent is given, we collect additional data, including feasibility measures (recruitment rate, data collection feasibility, study completion rate, and protocol adherence; the journal information collected is restricted to the duration from admission to the ED until 30 days after inclusion), clinical parameters (patient characteristics and demographics, such as date of birth, sex, name, and comorbidities [Charlson Comorbidity Index]), and any vital signs recorded as part of standard patient management during the stay in the ED (systolic and diastolic blood pressure, mean arterial pressure, heart rate, peripheral oxygen saturation, Glasgow Coma Scale score, and tympanic temperature). All fluids administered both orally and intravenously will be documented.

The time and date of admission and discharge will be collected for the first 30 days. The time and date of any readmission or death will be collected for the first 30 days. The time and date of insertion and removal of any peripheral cannula will be documented, as well as the reason for removal. Blood tests for sodium, chloride, potassium, and creatinine from admission until 48 hours after inclusion will be collected.

**Figure 2:**
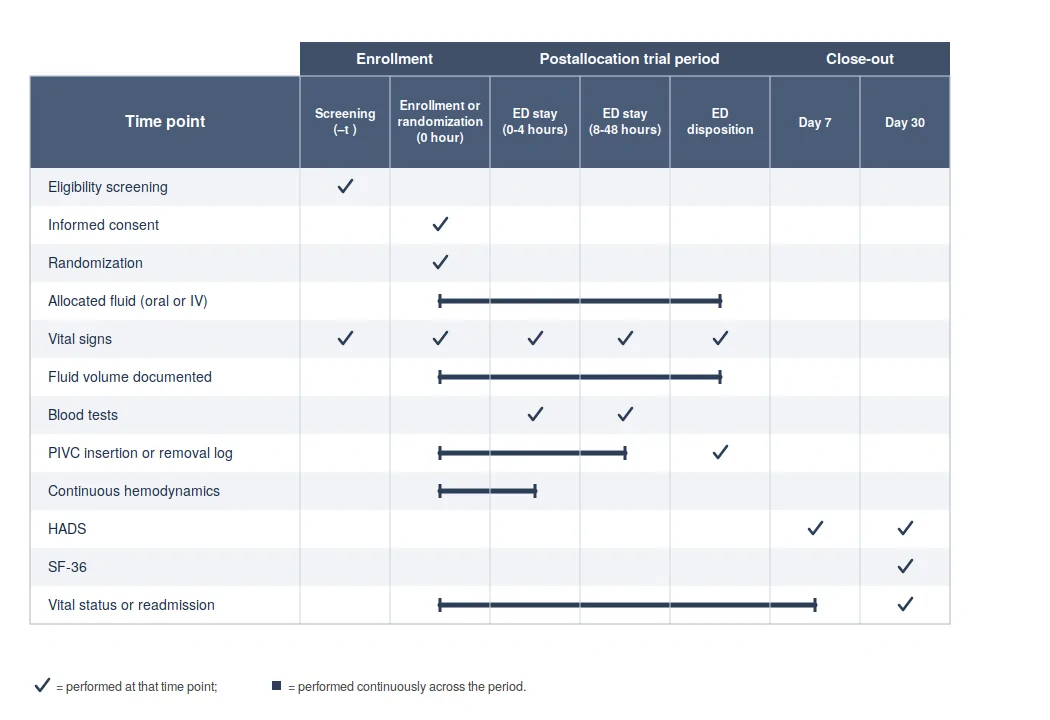
Schedule of enrollment, interventions, and assessments. Vital signs included systolic and diastolic blood pressure, mean arterial pressure, heart rate, peripheral oxygen saturation, Glasgow Coma Scale score, and tympanic temperature. Blood tests included sodium, chloride, potassium, and creatinine. Substudy 1 for continuous hemodynamics included 10 participants per arm and assessed blood pressure, cardiac output, stroke volume, pulse, and systemic vascular resistance during the first 4 hours. Substudy 2 for vital status and readmission included 10 participants per arm and assessed vital status, readmission, and hospital-free days through day 30. ED: emergency department; HADS: Hospital Anxiety and Depression Scale; IV: intravenous; PIVC: peripheral intravenous catheter; SF-36: 36-item Short Form Health Survey.

### Data Monitoring Committee and Trial Monitoring

No data monitoring committee has been established for this trial. The interventions involve administration of fluid therapy, which is routinely used as part of standard clinical care in the ED and considered a low-risk intervention. A data monitoring committee was deemed unnecessary. Trial oversight is instead provided by the local investigators responsible for trial conduct, participant safety, and protocol adherence.

### Data Analysis

Data will be stored for 10 years in accordance with Danish regulatory requirements. Data are stored in pseudonymized form and will be managed and analyzed using Stata (version 18; StataCorp) in collaboration with an independent biostatistician. We will not conduct any interim analyses. The primary analysis will be performed on both the intention-to-treat and per-protocol populations, as recommended for noninferiority trials. Missing data will not be imputed. Feasibility outcomes will be analyzed descriptively.

### Statistical Analysis

Data are stored in pseudonymized form for 10 years in accordance with Danish regulatory requirements and are managed and analyzed in R (version 4.6.1; R Foundation for Statistical Computing) in collaboration with an independent biostatistician blinded to allocation. No interim analysis is planned.

The primary analysis will be performed on both the intention-to-treat population (all randomized participants, regardless of the treatment received) and the per-protocol population (participants who completed the allocated route without a major protocol deviation). For a noninferiority trial, concordant results in both populations are required for the conclusion to be considered robust; a discordant result will be reported as inconclusive.

For the primary clinical outcome, let P_oral_ and P_IV_ denote the proportions achieving the prescribed volume in the 2 arms. Noninferiority will be assessed against the null hypothesis that P_IV_−P_oral_ is at least the noninferiority margin of 15 percentage points (absolute scale). The difference in proportions will be estimated with a 1-sided 97.5% CI (equivalent to a 2-sided 95% CI) using an exact proportion test (*prop.test* in R); noninferiority will be concluded if the lower confidence limit excludes a difference in favor of intravenous fluids of >15 percentage points.

Secondary binary outcomes will be compared using chi-square or Fisher exact tests, with relative risks and 95% CIs. Secondary continuous outcomes will be compared using independent *t* tests or Mann-Whitney *U* tests according to distribution, assessed by the Shapiro-Wilk test. Hospital-free days will be dichotomized as event-free at 30 days (yes or no) and analyzed as a binary outcome. Feasibility outcomes will be summarized descriptively. The planned presentation of the principal results is shown in Table 3.

**Table 3:** Template for presentation of the principal trial results.

Outcomes	Oral fluid therapy (n=125)	Intravenous fluid therapy (n=125)	Difference (95% CI)	*P* value
Primary outcome: achieved fluid volume, (%)	n (%)	n (%)	Risk difference (lower limit-upper limit)	*P*_NI_^a^
30-day mortality	n (%)	n (%)	RR^a^ (lower limit-upper limit)	*P*
Mean fluid received (mL)	Mean (SD)	Mean (SD)	Mean difference (lower limit-upper limit)	*P*
Acute kidney injury	n (%)	n (%)	RR (lower limit-upper limit)	*P*_AKI_^c^

^a^NI: noninferiority.

^b^RR: relative risk.

^c^AKI: acute kidney injury.

### Sample Size

A feasibility trial does not require a sample size calculation. The sample size is therefore determined by the primary clinical (noninferiority) outcome. No published data describe the proportion of adult ED patients who receive the full prescribed volume of fluids before disposition, and we identified no directly comparable benchmark. We expect 85% of patients in both arms to achieve the prescribed fluid volume. This assumption was established by structured consensus among the treating clinicians at the participating sites, informed by pediatric enteral-rehydration literature, in which failure of the oral route occurs in approximately 10% of children [26]. Estimating this proportion in adult ED patients is one of the objectives of the trial, and the observed proportion in the intravenous arm will be used to specify the sample size of any subsequent definitive trial. Under this assumption, for a binary outcome analyzing noninferiority of oral fluids, 240 patients in total are required with 90% power and a 1-sided α of .025 to exclude a difference in favor of the intravenous fluids of >15 percentage points. This margin was chosen based on clinical judgment that a difference of <15 percentage points would not be considered clinically relevant, given the advantages of oral administration. To accommodate dropouts, a total of 250 patients will be included.

### Ethical Considerations

The trial was registered at ClinicalTrials.gov (NCT07361952) on December 29, 2025, after recruitment had commenced on October 3, 2025. Registration was delayed unintentionally; the trial was approved by the Regional Committees on Health Research Ethics for the Region of Southern Denmark (S-20250042) on September 8, 2025. The processing of personal data was registered with the Region of Southern Denmark internal registry as required under the European Union General Data Protection Regulation and Danish Data Protection Agency regulations. Signed informed consent will be obtained for all participants after the study has been explained both in writing and orally. Only the sponsor and local investigators will have access to the final trial dataset. Other researchers may be granted access to the anonymized data for analysis upon reasonable request.

### Patient Involvement

No involvement of patients or the public in the design or reporting of the study has been planned.

### Risk and Side Effects

Overall, the risk to participants is minimal. All participants are prescribed fluids by a physician; the volume is not changed as part of the trial; oral fluids are safe and regularly used in clinical care for hydration. The only risk is the potential inability of certain patients to drink the amount of fluid prescribed by the attending physician within the defined time frame. But regular re-evaluation of the patient’s fluid intake, hydration status, and vital signs every 8 hours is part of the standard monitoring in the ED and will ensure that fluid management is adjusted by the attending physician if necessary. There is no evidence suggesting that oral fluid administration endangers patient safety, and intravenous fluid therapy remains available as a rescue option. Furthermore, the study does not restrict any other aspect of routine clinical care, including medications or additional fluid types. Participants in clinical trials are covered by the Danish patient compensation system administered by Patienterstatningen.

### Dissemination

The results of the study will be published in peer-reviewed scientific journals and disseminated through participation in academic conferences. Positive and negative results will be published according to the CONSORT (Consolidated Standards of Reporting Trials) statement for parallel-group randomized trials [31]. The author panel will include study members in accordance with the International Committee of Medical Journal Editors (ICMJE) criteria.

### Protocol Amendments

Protocol modifications will be communicated to the clinical trial registry and scientific ethics committee and described in future publications.

## Results

### Overview

The study was funded by the Region of Southern Denmark in May 2024. Recruitment for the trial began on October 3, 2025, at the participating EDs in Esbjerg, Odense, and Aabenraa. As of September 2026, a total of 85 patients have been randomized to 1 of the 2 treatment arms, corresponding to 34% of the target sample. The trial plans to include 250 participants in total. Recruitment is expected to be completed in October 2026, with final follow-up anticipated in November 2026. Results are expected to be published in summer 2027.

### Progress and Protocol Deviations

Enrollment has proceeded without any amendments to the eligibility criteria, intervention, outcome definitions, or statistical analysis plan. No serious adverse events attributable to the study intervention have been reported to the investigators. No interim analysis has been performed, and no comparative analysis of trial data has been undertaken; accordingly, no results of the primary or secondary outcomes are reported here.

## Discussion

### Principal Anticipated Findings

We anticipate that a randomized comparison of oral and intravenous fluid therapy can be delivered in routine emergency care and that most alert adults prescribed intravenous crystalloid will drink the prescribed volume when asked to. If protocol adherence is acceptable and the lower confidence bound for the difference in the proportion achieving the prescribed volume lies within 15 percentage points, a definitive effectiveness trial will be warranted. Two negative results would be equally informative. If clinicians decline to randomize eligible patients or if participants allocated to the oral route are frequently converted to intravenous administration, the obstacle lies in clinician equipoise rather than patient tolerance and must be addressed before a larger trial is attempted. If patients accept randomization but many cannot drink the prescribed volume, the definitive trial will require a narrower eligibility definition than the deliberately broad one used here. In either case, the trial will have identified the constraint that a larger trial must be designed around.

### Comparison With Prior Work

The physiological case for oral fluid therapy is more secure than its clinical evidence base. Water ingestion produces plasma volume expansion, splanchnic recruitment, peripheral vasoconstriction, and a rise in arterial pressure in healthy volunteers, in autonomic failure, and in shock [17-21], and our own crossover trial found a greater and more sustained rise in mean arterial pressure after oral than after intravenous fluid [22]. Clinically, enteral hydration has performed at least as well as the intravenous route wherever the 2 routes have been compared: in pediatric gastroenteritis, where a meta-analysis of 16 randomized trials reported shorter hospital stays and fewer serious adverse events [27]; in bronchiolitis [26]; in predicted severe acute pancreatitis [25]; and in the prevention of contrast-associated acute kidney injury [24].

But these settings are narrow, each concerning a single diagnosis, and several concern children. The adult ED is different, with an undifferentiated population in which the intravenous route is the default not because it is superior but because a catheter is easily available. This trial therefore tests not a physiological hypothesis but an assumption on which everyday practice rests and for which no direct evidence exists.

### Strengths

Site-stratified randomization removes the potential for confounding by site. The design is pragmatic and inclusive, with eligibility defined by the prescription rather than the diagnosis, physician discretion over fluid type, volume, and rate, no restriction on concurrent care, and intravenous rescue always available. The feasibility framework gives the trial a useful answer in either direction: it will enable a definitive trial or specify what must change before one can be run.

### Limitations

The trial is not powered for patient-centered outcomes, and no conclusion about mortality, organ dysfunction, or length of stay may be drawn from it; acute kidney injury and severe electrolyte derangement are exploratory safety signals, not tested hypotheses.

The intervention cannot be blinded, which may bias adherence; we mitigate this by blinding the analyzing statistician, prespecifying outcome definitions, and drawing volumes from routine nursing documentation. Oral intake is intrinsically less precisely measured than an infused volume, a limitation shared by all trials of enteral hydration; its extent is quantified through the data collection feasibility outcome and through the reporting of missingness, since the analysis is based on complete cases.

ED length of stay is a competing event that closes the observation window, and because oral administration is slower, early disposition may limit delivery in the oral arm more frequently.

The 15-percentage point margin was set by clinical consensus and is permissive: a noninferiority conclusion under it excludes only a large shortfall in fluid volume delivery. The assumed 85% baseline proportion is a working assumption, not an empirical estimate, as no published data describe it in adult ED patients; if the true proportion differs materially, the clinical comparison will be underpowered or overpowered, although the feasibility outcomes, being estimated descriptively, remain valid.

Selection bias arises from limited screening hours, the requirement for capacity to consent, and the treating clinician’s discretion to decline an approach. Consent is sought in an environment in which patients may feel unable to refuse.

Finally, the 3 Danish EDs located within a publicly funded system, and the deliberately heterogeneous cohort may limit generalizability and preclude identification of which diagnostic groups benefit; that question is deferred to the definitive trial.

### Future Directions

The outputs that matter most are design parameters: the proportion of adult ED patients who actually receive their prescribed volume by either route, the screening-to-randomization ratio, the frequency and reasons for crossover, and the catheter hours an oral strategy might avoid. We anticipate that the definitive trial’s primary end point should be patient-relevant rather than volume-related, since it is not in delivering the fluid that the oral route is hypothesized to confer benefit. A trial-based economic evaluation is a natural extension.

## Abbreviations

CONSORT: Consolidated Standards of Reporting Trials
ED: emergency department
ICMJE: International Committee of Medical Journal Editors
OPEN: Open Patient Data Explorative Network
OUH: Odense University Hospital
SPIRIT: Standard Protocol Items: Recommendations for Interventional Trials

## Data Availability

The data collected in this study are available from the corresponding author on reasonable request, in accordance with Danish data protection laws.
